# A latent profile analysis of tear cytokines and their association with severity of dry eye disease in the Dry Eye Assessment and Management (DREAM) study

**DOI:** 10.1038/s41598-024-51241-1

**Published:** 2024-01-04

**Authors:** Yineng Chen, Krishna Mallem, Penny A. Asbell, Gui-Shuang Ying

**Affiliations:** 1 Department of Ophthalmology, Perelman School of Medicine, University of Pennsylvania, Philadelphia, PA 19104 USA; 2 Drexel University College of Medicine, Philadelphia, PA 19104 USA; 3 University of Memphis, Memphis, TN 38152 USA

**Keywords:** Biomarkers, Medical research

## Abstract

This study is to identify subgroups of DED patients with different tear cytokine profiles and compare their DED symptoms and signs among subgroups. Baseline tear cytokines (IL-1β, IL-6, IL-8, IL-10, IL-17A, IFN-γ and TNF-α) were measured using a magnetic bead assay. DED symptoms were assessed by Ocular Surface Disease Index (OSDI) and signs were assessed by corneal and conjunctival staining, tear break-up time (TBUT), Schirmer’s test, tear osmolarity and meibomian gland dysfunction (MGD). Latent profile analysis was performed to identify subgroups, and their scores of DED symptoms and signs were compared using generalized linear regression. Among 131 patients with total tear volume > 4 µl from both eyes, subgroup 1 (n = 23) significantly higher in IL-6 and IL-8 (all p < 0.001) and subgroup 2 (n = 108) significantly higher in IL-10 (p = 0.03), IL-17A (p < 0.001), and IFN-γ (p < 0.001). Both subgroups were similar in demographics and DED symptoms, but subgroup 1 had significantly more severe DED signs: higher conjunctival staining (3.38 vs. 2.69, p = 0.04), corneal staining (4.26 vs. 3.03, p = 0.03), lower Schirmer’s test score (8.20 vs. 13.72, p < 0.001), and higher composite severity score of DED sign (0.62 vs. 0.45, p = 0.002). We identified two DED subgroups with different profiles of tear cytokines. Patients in these subgroups differed significantly in DED signs, supporting the inflammation’s role in DED development and progression.

## Introduction

Dry eye disease (DED) is a common ocular condition that affects a significant portion of the global population, with an estimated global prevalence of 5–50 percent^1^. In the United States, it impacts approximately 16.4 million adults, equivalent to 6.8 percent of the population^2^. DED is a multifactorial disease of the ocular surface, characterized by a loss of tear film homeostasis and a range of ocular symptoms^3^. Although commonly referred to as a single entity, it is a complex and heterogeneous disease with multiple causes and pathophysiological mechanisms, making its management challenging. The lack of consistent correlation between symptoms and signs also limits the effectiveness of current clinical guidelines^4^. Consequently, there is a growing interest in understanding the pathophysiology of DED and identifying biomarkers that could facilitate clinicians and scientists to examine, monitor, and treat the disease.

Ocular surface inflammation is believed to be involved in the etiology of DED, as evidenced by increased inflammatory cells and the response to anti-inflammatory drug treatments^5,6^. A Meta-analysis has demonstrated that DED patients exhibit higher levels of interleukin (IL)-1β, IL-6, IL-8, IL-10, interferon (IFN)-γ and tumor necrosis factor (TNF)-α compared to controls^7^. However, the cytokine profile within the DED population remains relatively unknown. A study analyzing cytokines in 131 tear samples from the Dry Eye Assessment and Management (DREAM) Study revealed weak but significant correlations between individual cytokines (IL-10, IL-17A, and IFN-γ) and DED signs^8^. Nevertheless, analyzing individual cytokines provides limited information considering the high correlation among cytokines and their multiple immune functions. Therefore, it is necessary to evaluate the cytokine profiles by simultaneously considering multiple cytokines to understand the roles of the cytokines in DED.

In this study, we employed a latent profile analysis (LPA) to classify DED patients into subgroups based on their tear cytokine profiles in terms of concentrations of IL-1β, IL-6, IL-8, IL-10, IL-17A, IFN-γ, and TNF-α by utilizing unique data from the multicenter DREAM Study (NCT02128763). We then evaluated whether the DED symptoms and signs differ among these identified subgroups based on tear cytokine profiles.

## Method

### Study design and participants

The details about DRAME study trial design and methodology have been reported in previous publications^9,10^, only the key features of the study that are related to this study are described here. In brief, the DREAM study is a multicenter, randomized, double-masked clinical trial that reported no difference between oral omega-3 (ω3) supplementation and placebo groups in symptoms and signs of DED^10^. The DREAM study protocol received approval from a centralized Institutional Review Board at University of Pennsylvania (IRB PROTOCOL#: 816490, Philadelphia, PA, USA). Written informed consent was obtained from all study participants. The study was conducted in compliance with the tenets of the Declaration of Helsinki and authorized by FDA as an Investigational New Drug (IND) application.

### Assessment for dry eye symptoms and signs

Ocular Surface Disease Index (OSDI) was used to measure dry eye symptoms. It is a 12-item questionnaire designed to assess the ocular irritation in DED and its impact on vision-related functioning over the past week. The questionnaire consists of 3 subscales: ocular symptoms, vision-related function, and environmental triggers, each rated on a 0–4 scale. A final score ranging from 0 to 100 was calculated for each subscale, with higher scores indicating more/worse symptoms^11^. For each patient, the OSDI total score was calculated as the average of 3 OSDI subscale scores.

DED signs measured for each eye included tear break-up time (TBUT), corneal staining score, conjunctival staining score, Schirmer’s test, meibomian gland dysfunction, and tear osmolarity. TBUT measures the number of seconds between the last blink and the first appearance of discontinuity in the tear film, with a shorter time indicating greater tear film instability. The average of three repeated TBUT measurements in each eye was used. Schirmer test performed with anesthesia measures the length of the moistened area of the paper strips placed in the lower eyelid in mm/5 min, with shorter lengths indicating less tear production. Ocular surface damages were evaluated by corneal fluorescein staining and conjunctival lissamine green staining, with a higher score suggesting more severe damage. Corneal fluorescein staining was graded using the National Eye Institute [NEI]/industry-recommended guidelines^12^ on a scale of 0–15 per eye. Lissamine green conjunctival staining was graded using a modified version of the NEI guidelines on a scale of 0–3 in the temporal and nasal section for a total possible score of 6 in each eye. Tear osmolarity was measured on a scale of 275–400 mOsm/L with a TearLab™ Osmolarity System (San Diego, California). Meibomian gland dysfunction (MGD) was evaluated for plugging and lid secretion on a scale of 0–3 using the TearScience Meibomian Gland Evaluator™ at slit lamps. The MGD score was calculated by taking the average scores for plugging and lid secretion. For both tear osmolarity and MGD, higher scores indicate a greater abnormality. A composite sign severity score was also calculated as described in previous studies^13^ by taking the mean of the severity scores of these six DED signs, with a higher score indicating a worse sign.

### Tear sample collection and cytokine analysis at baseline

The study protocol provided clear guidelines for study personnel at clinical sites to properly collect, store and ship tears samples. Following the standard operating procedure^14^, non-stimulated tears from both eyes were collected without anesthesia using microcapillary tubes at the lateral canthus at least two hours prior to ocular exam for dry eye signs. Before participants were randomized to treatment with omega-3 supplement or placebo at baseline, tear sample was collected from each eye within a 5-min period between 11:00 AM and 4:00 PM to reduce the impact of diurnal variation. Tears from the left and right eye were pooled since DED patients had low levels of basal tear production.

Tears transferred to a new precooled, prelabeled tube, with buffer added until a total volume of 50 µl^8,14^. The tear samples were transferred on dry ice to the ocular biomarker research lab at the Icahn School of Medicine at Mount Sinai and stored at − 80 °C freezers until analysis. All assays were performed within 12 months of collection.

Tear cytokine measurements have been described in detail in our previous publication^8^. In brief, concentrations of IL-1β, IL-6, IL-8, IL-10, IL-17A, IFN-γ, and TNF-α were measured by MILLIPLEX-MAG kit (High Sensitivity Human Cytokine Kit, Cat #HSCYTMAG-28SK, Millipore Corporation, Billerica, MA 01,821) as per the manufacturer’s manual and the laboratory standard operating procedures^14^. The kit specified minimal detectible cytokine concentrations were: IL-1β, 0.12 pg/ml; IL-6, 0.13 pg/ml; IL-8, 0.12 pg/ml; IL-10, 0.58 pg/ml; IL-17A, 0.28 pg/ml; IFN-γ, 0.32 pg/ml and TNF-α, 0.15 pg/ml. Sample cytokine with concentrations less than the minimal detectible concentrations were assigned the ‘0’pg/ml value in the statistical analysis. In addition, we performed a sensitivity analysis by imputing the concentration of cytokine level below the minimal detectible concentrations via the Multiplicative lognormal replacement method by the R package zCompositions^15^.

Among total of 535 DREAM participants from 27 sites across the United States, 244 were from 17 sites without − 80 °C storage capacity for tear sample, 58 did not provide tear samples at baseline visit, and 102 did not provide sufficient tear samples (at least 4 µl) for accurate cytokine measurement. Only 131 participants had ≥ 4 µl (pooled team sample from two eyes) at baseline were included in the statistical analysis.

A previous study of tear inflammatory cytokine concentrations among DED patients indicated 81% of DED patients could provide at least 4 μl pooled tears from two eyes by using our SOPs for team sample collection, and cytokine measurement results were highly reproducible and reliable with tear volumes ranging from 4 to 10 μl^14^. We previously reported that the cytokine concentrations assessed from pooled tear sample of at least 4 μl were significantly correlated with severity of DED signs, including conjunctival staining score, corneal staining score, TBUT, and Schirmer’s test score^8^.

### Statistical analysis

We performed latent profile analysis (LPA) to examine how the profiles of 6 tear cytokines jointly can be used to group DED patients based on their underlying constructs representing ocular inflammation. The LPA is a statistical clustering approach to uncover unobserved classes within a heterogeneous population, such as DED patients. Individuals within a subgroup exhibit a unique set of observed continuous characteristics—tear cytokine levels in our case—that are different from that of other subgroups^16^.

Determination of subgroups is achieved by calculating posterior probabilities, which indicate the likelihood of a subject belonging to a specific subgroup. Individuals are assigned to the subgroup with the highest probability^17^. Employing LPA to identify meaningful cytokines profile subgroups enables us to evaluate the association between cytokines and severity of dry eye disease. We fit a series of latent profile models, each differing in the number of profiles and covariance matrix: (1) Equal variances and covariances fixed to 0, (2) Varying variances and covariances fixed to 0, (3) Equal variances and equal covariances, (4) Varying variances and varying covariances^18^. We chose the best LPA models based on the Akaike’s Information Criteria (AIC) and Bayesian Information Criteria (BIC), entropy and adjusted Lo-Mendell-Rubin test. In the LPA analysis, the cytokine values were standardized by subtracting the mean from each original value and dividing it by the sample standard deviation. The LPA modeling was conducted using the R package tidyLPA version 1.1.0 and MplusAutomation Version 1.1.0.

The subgroups of DREAM participants identified from the best LPA model were compared for their demographics, baseline DED symptoms and signs. The linear regression models were used to compare continuous person-level characteristics, logistic regression models were used to compare binary and categorical person-level characteristics (demographics and dry eye symptoms). For the comparison of eye-specific DED signs between subgroups identified from latent profile analyses, generalized estimating equations were used to account for inter-eye correlation. The baseline tear cytokines were summarized using median (inter-quartile) and compared between subgroups of patients using Wilcoxon rank sum test due to the skewness of the cytokine data. Statistical comparisons between subgroups were performed in SAS v9.4 (SAS Institute, Inc., Cary, NC), and two-sided p < 0.05 was considered to be statistically significant.

## Results

This study included 131 participants with at least 4 µl of pooled tear sample from left eye and right eye at baseline. As reported previously patients with low tear volume (< 4 µl) were older and had worse dry eye signs^8^.

The baseline characteristics of these 131 participants including their baseline DED symptoms, signs and tear cytokine levels were summarized in Table 1. Their mean age was 54 years (range 20–82 years), approximately 80% were female, 59% were White, 6% had Sjogren’s syndrome. The baseline tear cytokine concentrations were summarized in Table 2. The percent of patients with tear cytokine concentration below detectable level ranged from 1.5% (IL-8) to 54.2% (TNF-α).

**Table 1:** Characteristics of participants for the latent profile analysis.

Baseline characteristics	
Person-level characteristics (N = 131 participants)	
Age (years): Mean (SD)	54.2 (14.1)
Gender: Female (%)	105 (80.2%)
Race (%)	
White	77 (58.8%)
Black	16 (12.2%)
Asian	8 (6.1%)
Other	30 (22.9%)
Sjogren syndrome: Yes (%)	8 (6.1%)
OSDI score: Mean (SD)	
Total	42.0 (15.3)
Environmental triggers subscale	54.2 (27.0)
Ocular symptoms subscale	43.6 (18.6)
Vision-related function subscale	35.1 (20.0)
Eye-lever characteristics (N = 262 eyes)	
Conjunctival staining score: Mean (SD)	2.8 (1.4)
Corneal staining score: Mean (SD)	3.2 (2.7)
Tear break-up time (seconds): Mean (SD)	3.3 (1.6)
Schirmer test score: Mean (SD)	12.7 (8.6)
Meibomian gland dysfunction: Mean (SD)	1.9 (1.0)
Tear Osmolarity (mOsm/L): Mean (SD)	300.5 (15.7)
Composite sign severity score: Mean (SD)	0.5 (0.3)

**Table 2:** Descriptive statistics of tear cytokine concentrations at baseline (N = 131 participants).

Tear cytokines (pg/mL)	Patients with cytokine below detectable level	Mean (SD)	Median (1st quartile, 3rd quartile)
IL-1β	29 (22.1%)	4.3 (5.0)	3.5 (1.1, 5.5)
IL-6	5 (3.8%)	14.3 (47.3)	5.4 (3.1, 9.5)
IL-8	2 (1.5%)	397 (1738)	33.8 (15.1, 96.2)
IL-10	37 (28.2%)	11.4 (13.7)	8.0 (0.0, 16.2)
IL-17A	28 (21.4%)	8.9 (8.6)	8.0 (1.6, 12.3)
IFN-γ	8 (6.1%)	32.3 (28.5)	27.7 (9.8, 41.4)
TNF-α	71 (54.2%)	3.0 (9.3)	0.0 (0.0, 3.5)

**Figure 1:**
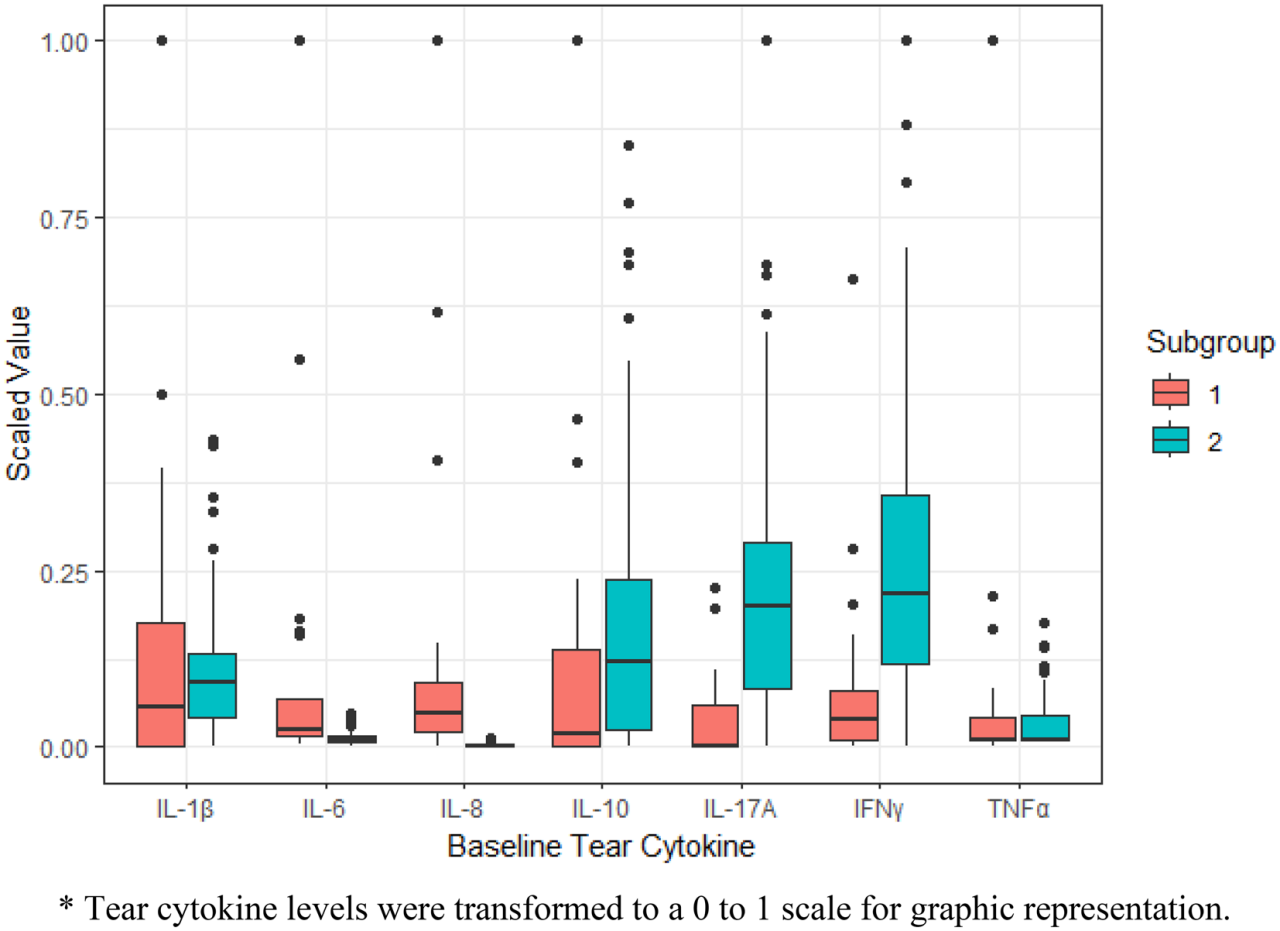
Tear cytokine levels in two subgroups of participants identified by latent profile analysis of baseline tear cytokines.

The statistics from various latent profile models were summarized in Table 3. The 3-profile model with variance and covariance vary across profiles had the lowest BIC. However, adjusted Lo–Mendell–Rubin likelihood ratio test showed an insignificant p-value for the 3-profile model (p = 0.70), indicating that the 3-profile model is not significantly better than the 2-profile model. This 2-profile LPA model yielded two subgroups of participants with mean posterior probability was 0.998 for subgroup 1 and 0.999 for subgroup 2. As shown in Fig. 1 and Table 4, subgroup 1 (n = 23) was characterized by significantly higher levels of IL-6 and IL-8 (all p < 0.001), while subgroup 2 (n = 108) was characterized by significantly higher levels of IL-10 (p = 0.03), IL-17A (p < 0.001), and IFN-γ (p < 0.001). There was no statistically significant difference between two subgroups in IL-1β (p = 0.44) and TNF-α (p = 0.94). The sensitivity analysis using imputed cytokine values for patients with cytokine values below detectable limit provided similar results.

**Table 3:** Goodness of fit for various latent profile analysis models of baseline tear cytokines.

Variances	Covariances	Number of Classes	AIC	BIC	Entropy	Adjusted LMR (p)*
Equal across classes	Fixed to 0					
		2	2372	2435	1.00	0.61
		3	2290	2376	1.00	0.13
Vary across classes	Fixed to 0					
		2	1164	1247	0.981	0.001
		3	686	813	0.989	-
Equal across classes	Equal across classes					
		2	1713	1837	1.00	0.08
		3	1528	1675	1.00	0.26
Vary across classes	Vary across classes					
		2	**365**	**569**	**0.998**	** < 0.001**
		3	119	418	0.986	0.70

Significant values are in [bold].

*p value of adjusted Lo-Mendell-Rubin test.

**Table 4:** Comparison of baseline tear cytokines between two subgroups of participants identified from latent profile analysis of baseline tear cytokines.

Cytokine (pg/ml)	Median (inter-quartile)		p-value*
	Subgroup 1 (n = 23 participants)	Subgroup 2 (n = 108 participants)	
IL-1β	2.2 (0, 7.0)	3.6 (1.6, 5.2)	0.44
IL-6	13.3 (7.6, 32.2)	4.4 (2.9, 7.6)	** < 0.001**
IL-8	677 (347, 1495)	28.8 (13.9, 58.6)	** < 0.001**
IL-10	1.4 (0, 9.6)	8.6 (1.6, 16.6)	**0.03**
IL-17A	0 (0, 2.8)	9.4 (3.9, 13.8)	** < 0.001**
IFN-γ	5.5 (1.6, 11.3)	30.9 (16.9, 50.7)	** < 0.001**
TNF-α	0 (0, 3.3)	0 (0, 3.5)	0.94

Significant values are in [bold].

*From Wilcoxon rank sum test.

As shown in Table 5, two subgroups of participants were similar in the distribution of age (p = 0.99), gender (p = 0.74), race (p = 0.41), Sjogren’s syndrome (p = 0.13), and DED symptoms (p = 0.50). However, subgroup 1 had significantly more severe DED signs than subgroup 2 in terms of higher scores of conjunctival lissamine staining [mean (SD): 3.4 (1.7) vs. 2.7 (1.3), p = 0.04] and corneal fluorescein staining [4.3 (2.3) vs. 3.0 (2.7), p = 0.03], lower scores of Schirmer’s test [8.2 (5.1) vs. 13.7 (8.9), p < 0.001], and higher composite severity score of DED signs [0.62 (0.27) vs. 0.45 (0.27), p = 0.002]. There is no significant difference in meibomian gland dysfunction score (p = 0.87) and tear osmolarity (p = 0.06).

**Table 5:** Comparison of baseline demographic, dry eye symptoms and signs between two subgroups of participants identified from latent profile analysis of baseline tear cytokines.

Baseline characteristic	Subgroup 1 (n = 23 participants, 46 eyes)	Subgroup 2 (n = 108 participants, 216 eyes)	p-value
Person-level characteristics			
Age (year): Mean (SD)	54.2 (13.2)	54.2 (14.3)	0.99
Gender: Female (%)	19 (82.6%)	86 (79.6%)	0.74
Race (%)			
White	14 (60.9%)	63 (58.3%)	0.41
Black	2 (8.7%)	14 (13.0%)	
Asian	3 (13.0%)	5 (4.6%)	
Other	4 (17.4%)	26 (24.1%)	
Sjogren syndrome: Yes (%)	3 (13.6%)	5 (4.9%)	0.13
OSDI score: higher is worse, Mean (SD)			
Total	40.0 (17.21)	42.4 (14.95)	0.50
Environmental triggers subscale	50.0 (26.76)	55.1 (27.07)	0.42
Ocular symptoms subscale	38.8 (19.88)	44.6 (18.20)	0.17
Vision-related function subscale	35.6 (20.84)	35.0 (19.86)	0.91
Dry eye signs: Mean (SD)			
Conjunctival lissamine staining score,higher is worse	3.38 (1.71)	2.69 (1.29)	**0.04**
Corneal fluorescein staining score: higher is worse	4.26 (2.33)	3.03 (2.70)	**0.03**
Tear break-up time (sec), lower is worse	3.01 (1.11)	3.35 (1.65)	0.14
Schirmer test (mm), lower is worse	8.20 (5.14)	13.72 (8.89)	** < 0.001**
Meibomian gland dysfunction, higher is worse	1.97 (0.99)	1.94 (1.03)	0.87
Tear osmolarity (mOsms/L), lower is worse	296.95 (9.40)	301.28 (16.70)	0.06
Composite dry eye severity score*: higher is worse	0.62 (0.27)	0.45 (0.27)	**0.002**

Significant values are in [bold].

*The composite dry eye severity score, ranging from 0 to 1 was calculated from tear break-up time, anesthetized Schirmer’s test, corneal and conjunctival staining, and meibomian gland dysfunction.

## Discussion

Previous studies that have analyzed tear cytokine concentrations in DED have been limited to determining whether individual cytokines were elevated in DED^7,8^. By performing LPA that simultaneously consider multiple tear cytokines from DREAM participants with moderate-to-severe DED, we identified two subgroups of DED patients with different cytokine profiles. Subgroup 1 had significantly higher levels of IL-6 and IL-8, whereas subgroup 2 had significantly higher levels of IL-10, IL-17A and IFN-γ. These two subgroups of patients showed different severity of dry eye signs. Subgroup 1 had more severe DED signs than subgroup 2, including a greater conjunctival lissamine staining score and corneal fluorescein staining score, a lower Schirmer test score, and a greater composite severity score of dry eye signs. However, these two subgroups reported similar dry eye symptoms.

Roy et al. performed an analysis of individual baseline tear cytokine concentrations in the DREAM participants^8^. Similar to our study, they did not find any significant association between individual cytokine and DED symptoms. However, they did find associations between DED signs and certain individual tear cytokines. Specifically, they found that higher concentrations of IL-17A, IL-10, and IFN-γ were associated with lower corneal staining, while higher concentration of IFN-γ was also associated with lower conjunctival staining score. These findings correspond with our subgroup 2 that was identified through latent profile analysis. In addition, through LPA, we found subgroup 2 also had a higher Schirmer test score, supporting less severe dry eye signs in subgroup 2 patients. Our analysis differed from Roy et al.’s in that LPA also demonstrated an association between IL-6 and IL-8 with dry eye signs and found that both cytokines are elevated in subgroup 1 patients who had more severe dry eye signs than subgroup 2. Furthermore, LPA also showed that higher levels of IL-6 and IL-8 in subgroup 1 patients was associated with a greater composite dry eye severity score of signs. Our LPA was able to identify two different patterns of cytokine expression with meaningful differences in DED signs, which is a key difference between our own paper and Roy et al.’s.

Our analysis identify differential patterns of cytokine expression in DED patients. One meta-analysis of the existing literature on tear cytokine concentrations in DED patients found that all five cytokines (IL-6, IL-8, IL-10, IL-17A and IFN-γ) that differed significantly between two subgroups identified from our LPA had been shown to be elevated in patients with DED relative to controls^7^. Our LPA analysis found that IL-6 and IL-8 were higher in subgroup 1 that had more severe dry eye signs, while IL-10, IL-17A, and IFN-γ were higher in subgroup 2 that had less severe dry eye signs. These results further support prior findings and provide more evidence that inflammation plays an integral role in the pathogenesis of DED. Our findings also suggest that the underlying inflammation in DED is likely heterogenous in nature, and that there may be differential cytokine profiles producing differential DED disease subtypes. Given that DED itself is an incredibly heterogenous disease, these findings are not inherently surprising, but are of significance because associations between DED severity and specific cytokine profiles have not previously been identified.

While we cannot define specific immunologic pathways that contribute to various subtypes of DED using our results, it’s still useful to consider their potential implications. Cytokines are known to have broad pro and anti-inflammatory effects that can differ for each cytokine based on the specific pathway that is involved. Each of the five cytokines that varied significantly between subgroups have acknowledged roles in the immune response. IL-6 is a pro-inflammatory cytokine involved in the humoral-mediated adaptive immune response. IL-8 serves as a chemoattractant for inflammatory cells and is important in the innate immune response. IL-17A has a pro-inflammatory effect and is involved in increasing and coordinating the innate immune response. IFN-γ is integral in promoting cell mediated adaptive immunity. IL-10 has anti-inflammatory effects and helps downregulate the adaptive immune response^19–21^. Subgroup 1 was found to have significantly greater levels of IL-6 and IL-8, which suggests that both innate and humoral-mediated adaptive immunity are involved in the pathogenesis of DED in these patients. Given that patients in this subgroup were also found to have more severe DED signs, it’s also possible that there is a greater overall degree of inflammation in these patients, resulting in these worse DED signs. Subgroup 2 was found to have significantly greater levels of IL-17A, IL-10, and IFN-γ, with less severe DED signs. The increased levels of IL-10 potentially suggest a greater anti-inflammatory mechanism in subgroup 2 patients. The association of IL-17A and IFN-γ similarly suggests that the innate immune system and cell-mediated adaptive immunity are involved in pathogenesis of DED in subgroup 2 patients, and that an interplay of IL-17A, IL-10, and IFN-γ may result in a higher anti-inflammatory profile in these patients. As stated previously, cytokines can have multiple different effects, and while our results may be useful in theorizing different potential immunological bases for two subgroups of DED participants, our interpretations are limited. We must also acknowledge that even though subgroup 1 and subgroup 2 varied in DED signs, there was no significant difference in DED symptom severity in terms of OSDI score, which further limits the utility of interpreting our findings in this manner.

There are several limitations with this study. Our sample only included 131 patients who provided sufficient volume of tear samples, which may have limited our capability of finding finer subgroups of DED patients and limit our statistical power to detect differences between subgroups, either in DED signs or symptom severity. Furthermore, because at least 4 µl of tear volume is required for accurate cytokine measurements, and patients with low tear volume (< 4 µl) were found to be older and had worse dry eye signs, our analysis have excluded patients with more severe disease who would not have produced a sufficient volume of tears for analysis. As such, we may have missed identifying another potential subgroup of DED patients. This may limit the generalization of our findings. In addition, the latent profile analysis assumes a mixture of Gaussian distributions of tear cytokine. However, the concentrations of tear cytokines in our study exhibited positive skewness, which may be attributed to the combination of multiple normal distributions. Tihomir and Bengt^22^ introduced a mixture model capable of accommodating non-normally skewed distributions that requires large sample size. Due to the limited sample size of the study, we cannot apply this modelling approach to our data. Future studies with larger and heterogenous patient population would help validate our findings and potentially elucidate more differences in cytokine profiles between subgroups of DED patients.

In summary, our latent profile analysis of tear cytokines provides further support to the role of inflammation in the pathogenesis of DED and suggests that inflammation in DED is not a homogenous process. The finding of two different potential subgroups of DED patients with key differences in DED signs suggests that there are likely multiple cytokine profiles that influence the pathogenesis of DED, and that clinically variable DED subgroups may eventually be identifiable based on tear cytokine concentrations.

## Data Availability

Data is available at https://hyperprod.cceb.med.upenn.edu/dream_download/index.php.
